# A Theoretical Study of Armchair Antimonene Nanoribbons in the Presence of Uniaxial Strain Based on First-Principles Calculations

**DOI:** 10.1021/acsaelm.3c00686

**Published:** 2023-07-26

**Authors:** Arash Yazdanpanah Goharrizi, Ali Molajani Barzoki, Siegfried Selberherr, Lado Filipovic

**Affiliations:** † Department of Electrical Engineering, Shahid Beheshti University, Tehran IR19395, Iran; ‡ Institute for Microelectronics, Technische Universität Wien, 1040 Wien, Austria; § CDL for Multi-Scale Process Modeling of Semiconductor Devices and Sensors at the CD0509, 1040 Vienna, Austria

**Keywords:** 2D materials, antimonene, nanoribbons, compressive and tensile strain, bandstructure, density of states

## Abstract

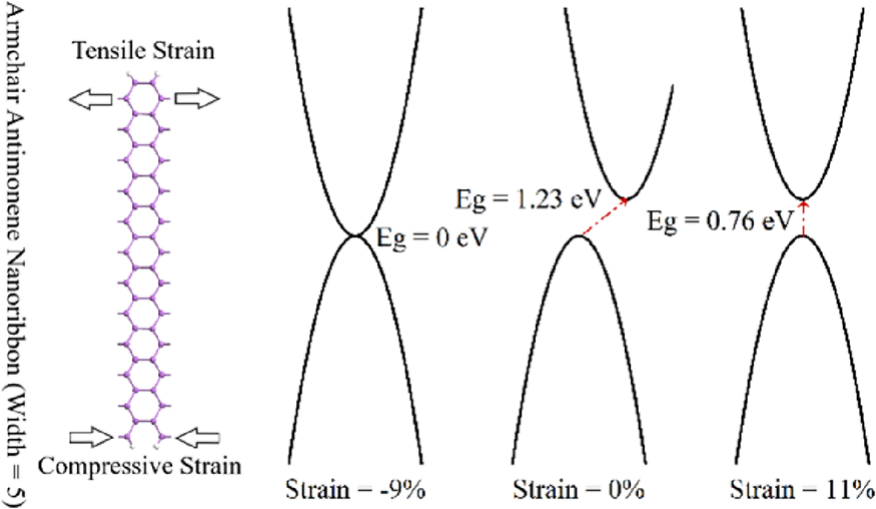

The optimized geometry and also the electronic and transport properties of passivated edge armchair antimonene nanoribbons (ASbNRs) are studied using *ab initio* calculations. Due to quantum confinement, the size of the bandgap can be modulated from 1.2 eV to 2.4 eV (indirect), when the width is reduced from 5 nm to 1 nm, respectively. This study focuses on nanoribbons with a width of 5 nm (5-ASbNR) due to its higher potential for fabrication and an acceptable bandgap for electronic applications. Applying uniaxial compressive and tensile strain results in a reduction of the bandgap of the 5-ASbNR film. The indirect to direct bandgap transition was observed, when introducing a tensile strain of more than +4%. Moreover, when a compressive strain above 9% is introduced, semi-metallic behavior can be observed. By applying compressive (tensile) strain, the hole (electron) effective mass is reduced, thereby increasing the mobility of charge carriers. The study demonstrates that the carrier mobility of ASbNR-based nanoelectronic devices can be modulated by applying tensile or compressive strain on the ribbons.

## Introduction

As can be evidenced from recent progress in the fabrication and realization of two-dimensional (2D) materials with extraordinary properties, such as a tunable bandgap size and high mobilities, these materials are promising candidates for future optoelectronic and nanoelectronic applications.^1−3^ Graphene, the best known and earliest-discovered 2D material, which is composed of a single layer of graphite, was introduced in 2004 by Novoselov *et al.*(4) Although having excellent electronic properties, such as high electron and hole mobilities, high thermal conductivity, and interesting optical properties, graphene is a gapless material.^5^ Many researchers have been investigating means to open the bandgap in graphene, such as through the fabrication of graphene nanoribbons (GNRs), which are one-dimensional (1D) materials.^6,7^ The bandgap of GNRs is inversely proportional to the width of the generated ribbons. After the discovery of graphene, other 2D materials, such as silicene, germanene, molybdenum disulfide (MoS_2_), phosphorene, arsenene, and antimonene have been realized and investigated.^8−11^ In addition to their unique properties, 2D materials of group V elements, unlike graphene sheets, have an intrinsic bandgap, making them more promising candidates for future nanoelectronic devices. Recently, several theoretical studies have focused on the geometric, optical, and electronic properties of phosphorene, arsenene, antimonene, and bismuthene.^11−15^ Several research groups have successfully synthesized 2D materials of group V elements using exfoliation or growth on different substrates.^16−20^ The ability to synthesize these films increases their potential for a wide range of applications from electronic, optoelectronic, and spintronic devices to sensors and actuators; further potential applications include thermoelectrics, energy conservation, and storage devices.^21−26^

The focus of this manuscript is antimonene, which has two allotropes named α (puckered) and β (buckled), which are very similar to black and blue phosphorene.^27^ Based on previous publications, the fundamental bandgaps of α- and β-antimonene sheets are about 0.54 eV and 1.18 eV, respectively.^28^ Although both α-phase and β-phase antimonene have semiconducting properties, they differ in their thermodynamic stability. β-phase antimonene has been shown to have a higher stability than α-phase antimonene, which makes it more suitable for various applications.^29−32^ Most of the newly discovered properties of antimony are obtained by restricting its structure to two dimensions and creating a 2D sheet structure. By applying further confinement to the sheet structure, antimonene nanoribbons can be achieved. Furthermore, in nanoribbons, the bandgap of 2D antimony can be tuned by varying the width.^33^ Therefore, without changing the material and without complex doping, antimonene nanoribbons can be applied to form heterostructures, which are widely utilized in electronic and optoelectronic applications.^34^ Moreover, the electronic properties of nanoribbons can be adjusted by a variety of methods, one of which is applying strain to the structure.^14,35−37^

In the present work, the effect of strain on the electronic and transport properties of β-antimonene nanoribbons with armchair edges and a width of 5 nm is theoretically investigated. We focus on the ASbNR structure with a width of 5 nm (5-ASbNR) because its bandgap size is sufficient for electronic applications, while technologically being the most likely to be realized, as 5 nm structures can readily be patterned. The hole and electron effective masses at the edges of the valence and conduction bands of 5-ASbNR are calculated at different applied strains. It is known that applying strain to the structure affects the mobility of electrons or holes, so we investigate the impact of compressive and tensile strain on the feasibility of realizing of p-type and n-type materials, respectively. In Section 2, the computational methods used in this study are described. Subsequently, in Section 3, the simulation results are presented and discussed. Finally, concluding remarks are provided in Section 4.

## Computational Methods

All first-principles calculations used in this study are performed in the framework of density functional theory (DFT) using the SIESTA package.^38^ The Fritz-Haber-Institute (FHI) pseudopotentials were used to describe the interaction between the valence and core electrons with a double zeta polarized basis set.^39^ We have used the Perdew–Burke–Ernzerhof (PBE) parametrized exchange-correlation functional within the generalized gradient approximation (GGA).^40−42^ The choice of pseudopotential and exchange-correlation functional have already shown to be accurate in representing the desired material, antimony, in previous studies.^33^ The cutoff energy was set to 105 Hartree to ensure convergence to the minimum total energy and the *k*-point sampling in the Monkhorst–Pack grid was chosen as 13 × 1 × 1 for general calculations and relaxation. To calculate the electronic band structure and the density of states (DOS), the *k*-point sampling was set to 21 × 1 × 1. A vacuum of 15 Å is employed along the vertical *z* axis to avoid any interaction between the two periodic layers.

To simulate transport, we model the device system using the non-equilibrium Green’s function (NEGF) formalism. This treats the system as a finite central (C) region, which is sandwiched between two semi-infinite left (L) and right (R) electrodes. To describe the scattering in the device, we can use Green’s function for the central region, given by
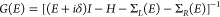
1where δ is a very small quantity and *H* is the channel region’s Hamiltonian. Σ_*L*_(*E*) and Σ_*R*_(*E*) are the contact self-energy functions, which describe how the central region is coupled with the left and right electrodes, respectively. We can calculate these using
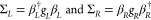
2where *g_L_* and *g_R_* are the left and right contacts’ surface Green functions, while β_*L*_ and β_*R*_ are the matrices that couple the device with the corresponding contact. The transmission probability of carriers through the channel can then be calculated using
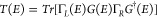
3where Γ is the contact-broadening function defined as
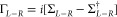
4

The NEGF approach for calculating carrier transport is coupled with *ab initio* DFT methods to obtain an accurate atomistic representation of charge carrier transport at the nanoscale, studied here.^43^

## Results and Discussion

**Figure 1:**
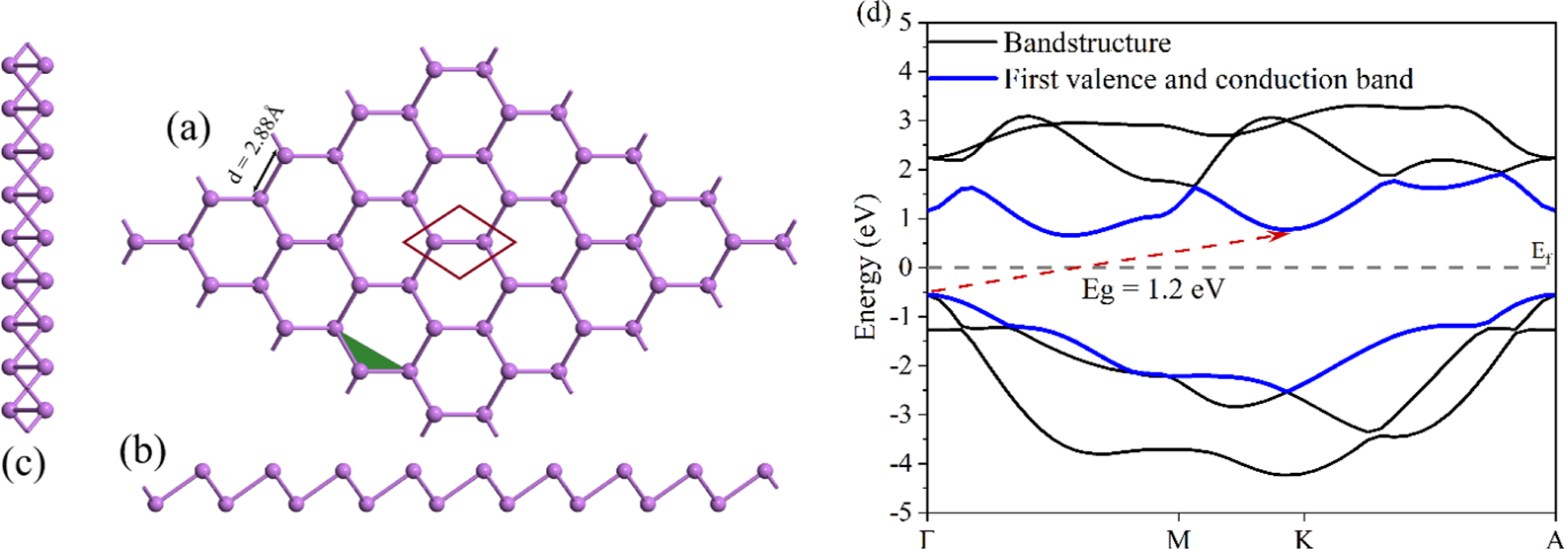
Structure of a β-allotrope antimonene sheet in its (a) top view and (b, c) side views. (d) Indirect band structure of the antimonene sheet, showing an energy bandgap of 1.2 eV. The Fermi energy level (*E*_f_) is set to 0 eV.

In order to verify the accuracy of the calculations carried out in this work, the relaxed configuration of the antimonene sheet (β-allotrope) along with its band structure is obtained and illustrated in Figure 1. The lattice constant, bond length, and angle between antimony atoms are obtained as 4.06 Å, 2.88 Å, and 89.98°, respectively.

According to the calculated band structure depicted in Figure 1c, the highest occupied states appear in the middle region, along the Γ-M line, while the lowest unoccupied states are located at the Γ point, which reveals an indirect energy bandgap of approximately 1.2 eV. Therefore, antimonene is veritably a semiconductor with an indirect energy bandgap. As can be seen from the simulation data presented in the Table 1, all the geometrical parameters we calculated for antimonene are roughly in agreement with previous theoretical and experimental studies.^18,21,28,43,44^

**Table 1:** Geometrical Parameters of ASbNRs, Bond Length, and Angle between Antimony Atoms along with the Bandgap of the Antimonene Sheet

		*a* = *b* (Å)	bond length *d* (Å)	angle θ(°)	buckling size Δ (Å)	bandgap *E*_g_ (eV)
theorical studies	this study	4.06	2.88	89.97	1.67	1.20
	(44)	4.04	2.87	89	1.67	1.04
	(45)	4.07	2.84	91.47	NA	1.37–1.99
experimental data	(18)	4.31	NA	NA	1.62	NA
	(46)	4.28	2.93	94	1.57	NA

**Figure 2:**
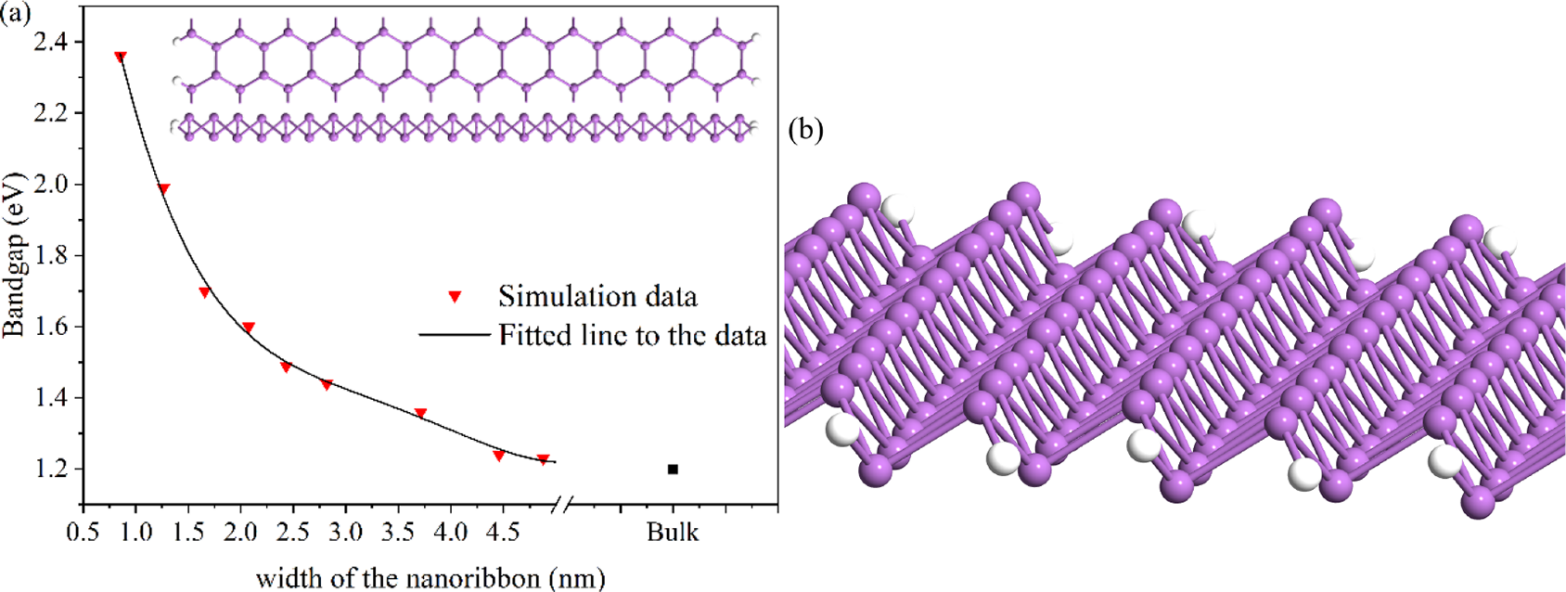
(a) Change in the energy bandgap with respect to the width of the nanoribbon, compared to the bandgap of the antimonene sheet. (b) Geometric structure of ASbNR.

The antimonene sheet can be cut along different directions, armchair or zigzag, to create a one-dimensional structure, which is referred to as armchair nanoribbons.^33^ In the present study, the electronic and transport properties of the β-allotrope of antimonene nanoribbons with armchair-shaped edges on both sides are investigated. The relaxed structure of armchair antimonene nanoribbons (ASbNRs) is shown in Figure 2a, where the edge atoms are passivated with hydrogen atoms. To obtain thermodynamic stability of the ASbNR sheets, the cohesive energies related to different widths were calculated using^47^
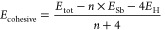
5where *E*_tot_, *E*_Sb_, *E*_H_, and *n* are the total energy of the nanoribbons, the energy of antimony atoms, the energy of hydrogen atoms, and the number of the antimony atoms in the unit cell of ASbNRs, respectively. These energies are calculated after the structures have been permitted to relax. Based on the obtained cohesive energies for different widths, it can be confirmed that all nanoribbons are thermodynamically stable. Wider ribbons are nevertheless more stable, as noted in the results provided in Table 2, showing that reducing the width of the nanoribbon increases the cohesive energy, making the ribbon more difficult to form. Similar to graphene nanoribbons, it is expected that the energy bandgap of an ASbNR sheet scales with the width of the nanoribbons.^33^ The energy bandgap of ASnNR as a function of ribbon width is calculated and plotted in Figure 2a. Our calculations show that the ASnNRs are semiconductors with energy bandgaps, which decrease as a function of increasing ribbon widths, which is consistent with previous studies.^33^ Quantum confinement plays an important role in opening the energy bandgap of ASnNRs, whereby the energy bandgap is found to be inversely proportional to the width. Due to the quantum confinement effect, the energy bandgap varies from 1.2 eV to 2.4 eV when the ribbon width is reduced from 5 nm to 1 nm, respectively.

**Table 2:** Cohesive Energies for Different Widths of ASbNRs as a Thermodynamic Stability Metric

width (nm)	*E*_cohesive_ (eV/atom)
0.854	–1.7065
1.263	–1.8598
1.658	–1.9379
2.075	–1.9861
2.43	–2.0189
2.819	–2.0424
3.713	–2.0741
4.46	–2.0898
4.87	–2.0976

**Figure 3:**
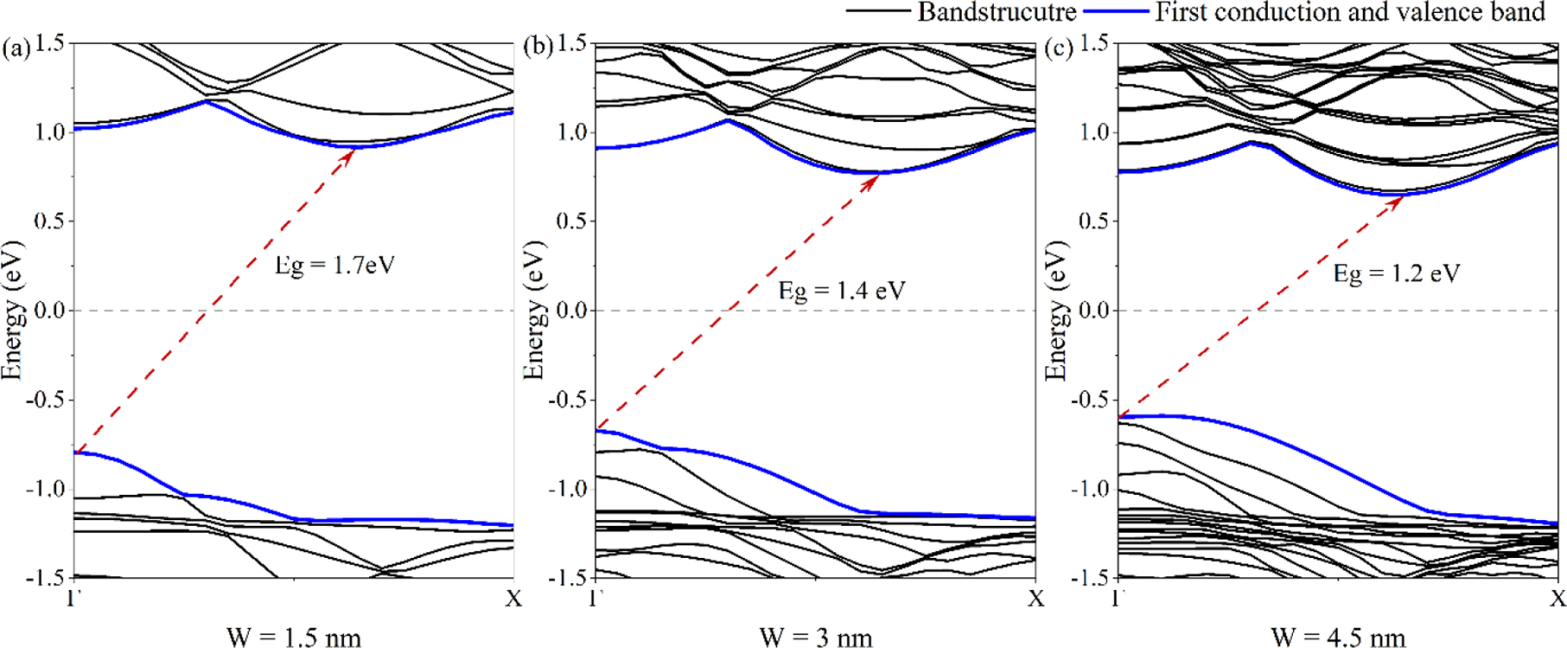
Band structure of ASbNRs at different widths: (a) 1.5 nm, (b) 3 nm, and (c) 4.5 nm. The dashed line indicates the Fermi level (gray line).

The band structure of ASbNRs for different widths (1.5 nm, 3 nm, and 4.5 nm) is calculated and plotted in Figure 3. According to the band structure, the ASbNRs are indirect semiconductors, where the valence band maximum (VBM) is located at the Γ point and the conduction band minimum (CBM) is located between the Γ and X points.

Based on the simulation results, the bandgap size of ASbNRs at a width of 5 nm is about 1.2 eV, while the bandgap does not change significantly when increasing the widths beyond 5 nm. Since the bandgap size at a width of 5 nm is suitable for designing electronic devices (close to the energy bandgap of silicon), the width of the ribbon is established at 5 nm for the remainder of this study, and we refer to this design as 5-ASbNR (5 nm armchair antimonene nanoribbon). Furthermore, from the technological feasibility and thermodynamic stability point of view, fabricating 5 nm nanoribbons is easier than narrower ones.^18,19^

The electronic properties of nanosheets and nanoribbons can be tuned using a variety of different methods. One of the very effective methods includes applying strain to the nanostructure.^37^ In the present work, the electronic properties of 5-ASbNR, such as its bandgap and the effective mass of charge carriers (electrons in the conduction band and holes in the valence band), are investigated after applying uniaxial strain.

Here, we applied uniaxial strain in the expansion direction of the 5-ASbNR, and the amount of strain is calculated as a percent change in the length of the unit cell using
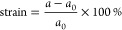
6where *a* and *a*_0_ are the lengths of the strained and unstrained unit cells, respectively.

**Figure 4:**
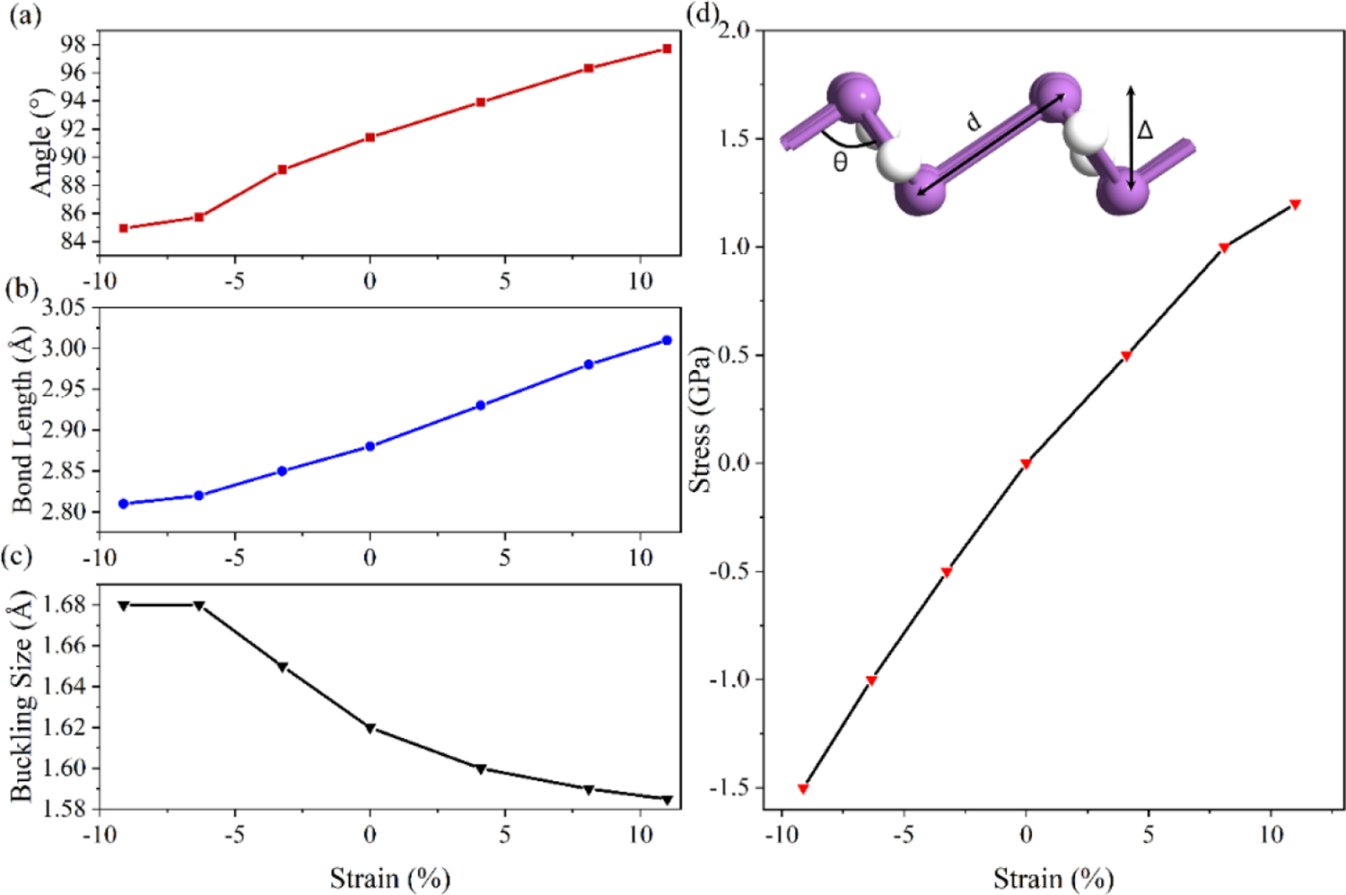
Strain-dependent changes in the geometrical parameters of ASbNRs, such as the atomic angle (a), are presented. (b) Variation of the bond length (c) and the buckling size (d) of ASbNRs with strain is also demonstrated. (d) Stress–strain curves are used to assess the stiffness of the nanoribbon.

To evaluate the stiffness of 5-ASbNR under strain, the strain–stress curves were computed and plotted in Figure 4d. The curve shows a linear relationship between stress and strain, without reaching a peak value at the fracture point, where the curve should drop sharply. The fracture point corresponds to the ultimate strength and strain, which indicate the maximum stress and deformation that the materials can withstand before breaking. The results reveal that 5-ASbNR does not experience the fracture point in the strain range of −9% to 11%, demonstrating the ribbon’s stability.^37^ To provide more clarity, the geometrical parameters of 5-ASbNR such as the angle between atoms (θ), bond length (*d*), and the buckling size (Δ) under different strains were calculated and presented in Figure 4a–c.

**Figure 5:**
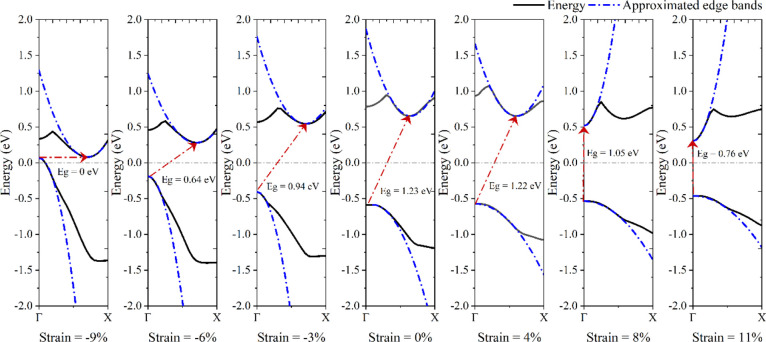
Conduction and valence band edges of ASbNR with a width of 5 nm under uniaxial compressive and tensile strain between −9% and 11%. The fitted curves show the approximated band edge with the charge carrier effective mass. The dashed-dotted lines indicate the Fermi level (gray line).

In the following, the effect of uniaxial compressive and tensile strain between −9% and +11% on the conduction and valence band edges of 5-ASbNR is investigated, cf. Figure 5.

The approximated band structure of nanoribbons, according to a quadratic fit to the CBM and VBM, is obtained and plotted in Figure 5 at various applied uniaxial strains; the curvature gives the electron (*m*_e_^*^) and hole (*m*_h_^*^) effective mass as follows
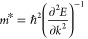
7where *E* and *k* correspond to the energy and the reciprocal lattice vectors along the nanoribbon, *ℏ* is the reduced Planck constant, and m* is the effective mass of electrons/holes (i.e., *m*_e_^*^/*m*_h_^*^).

**Figure 6:**
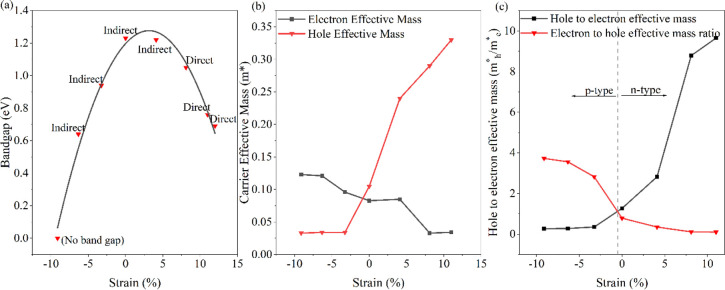
Effect of compressive and tensile strain on the (a) bandgap and (b) charge carrier effective mass of ASbNR with a width of 5 nm. (c) Ratios of the effective mass of holes to that of electrons or vice versa, i.e., *m*_e_^*^/*m*_h_^*^ or *m*_h_^*^/*m*_e_^*^, respectively.

Under no applied strain, the 5-ASbNR is an indirect semiconductor, where the CBM is located between the Γ and X points (close to the X point) and the VBM is at the Γ point. Furthermore, the effective mass of the holes is slightly larger than that of the electrons. By applying compressive strain, however, both the CBM and the VBM move toward the Fermi energy, but the location of the *k* points remains similar to that of the pristine ribbon. Therefore, the bandgap size of 5-ASbNR is decreased while remaining indirect. At a strain of −9%, a semi-metallic behavior can be observed. By applying compressive strain, the effective mass of electrons in the ribbon becomes larger than the effective mass of holes. Compared to the pristine (unstrained) structure, by applying tensile strain to 5-ASbNR, the location of the CBM in the *k* space moves toward the Γ point, while the location of the VBM remains unchanged at the Γ point. In addition, the stabilization of the CBM occurred by moving to lower energies. Therefore, the bandgap size is decreased by tensile strain, and the direct bandgap is observed for strains greater than +4%. Compared with the pristine structure, the effective mass of electrons is decreased, while the effective mass of holes is increased, when applying tensile strain. For a better comparison, the effect of strain on the bandgap and the effective mass of the charge carriers inside the ribbons are demonstrated in Figure 6. According to the results, by applying a tensile strain above 4%, the ASbNRs can be used for optoelectronic applications.

To evaluate the transport properties of 5-ASBNR under uniaxial strain, we first implemented a simple model to discuss the effect of the effective mass on the carrier mobility. Then, we used the non-equilibrium Green’s function (NEGF) formalism to study the transport property of 5-ASbNR.

Assuming that the carriers have random velocities after each scattering event, we can use a simple model to analyze the effect of the effective mass on the carrier mobility.^48^
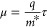
8τ, *q*, and m* are the scattering time, the elementary charge, and the effective mass, respectively. In the case of large applied strains, the scattering time may change relative to the applied strain value, but the variation in the applied strain in the present work is moderate. As a result, it can be assumed that the scattering time for the pristine and strained nanoribbons is roughly the same. Therefore, it is possible to evaluate the increasing or decreasing rate of mobility in nanoribbons according to the variation in the strain. Since the carrier mobility is inversely proportional to the effective mass, cf. eq 8, the hole and electron mobilities are increased and decreased, respectively, when compared with the pristine 5-ASbNR, after applying compressive strain; the opposite behavior is observed in the presence of tensile strain. According to the obtained results, it can be concluded that the unstrained and tensile-strained 5-ASbNR are suitable for n-type applications, while the ribbon is suitable for p-type applications in the presence of compressive strain.

**Figure 7:**
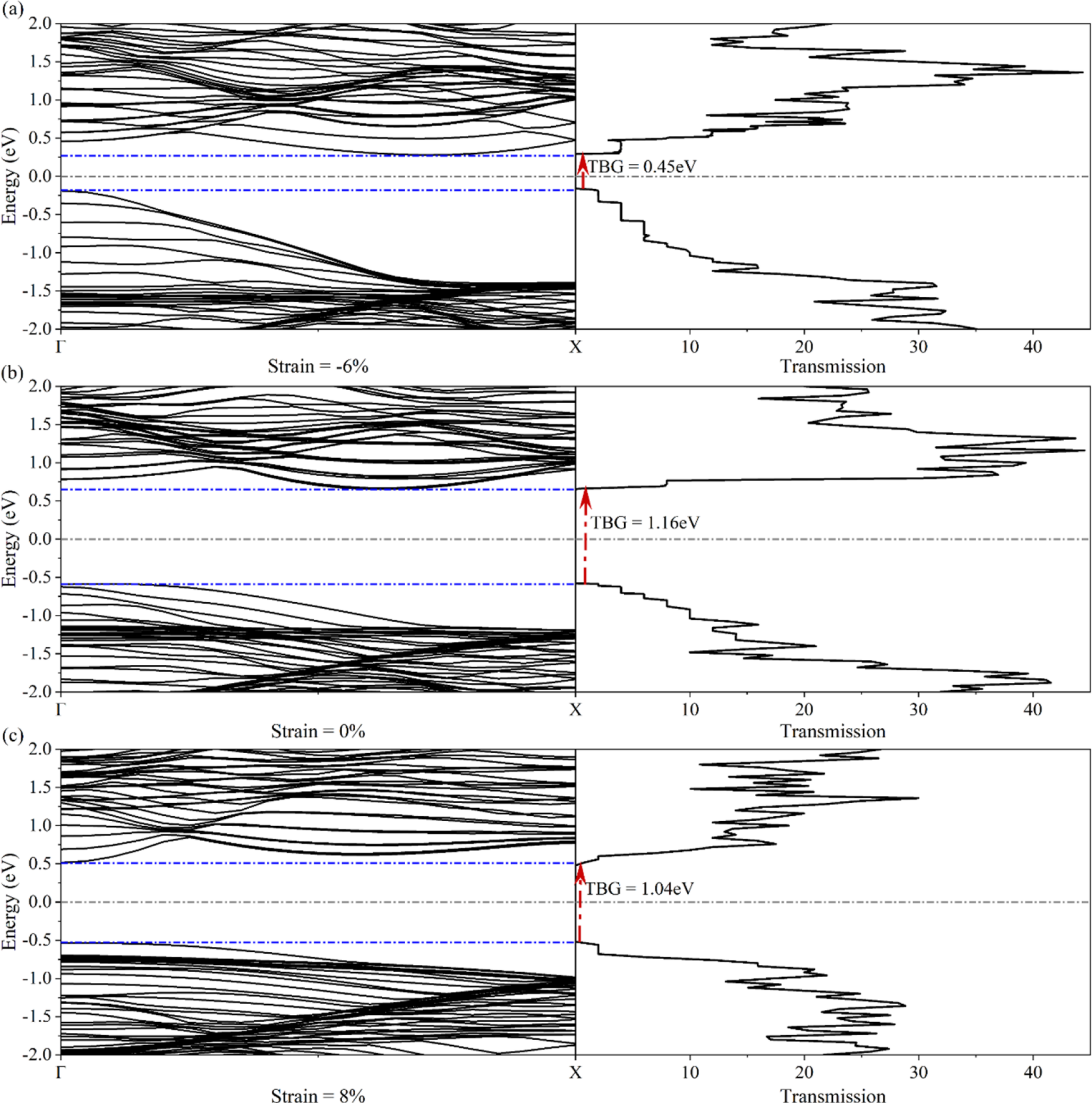
Transmission and band structure of 5-ASbBR for different applied strains, i.e., (a) −6%, (b) 0%, and (c) 8%. The dashed-dotted line indicates the Fermi level (gray line).

For a deeper understanding of the transport properties of 5-ASbNR in the presence of strain, the transmission spectrum of carriers at a length of 10 nm for −6%, 0%, and 8% strained ribbons is calculated using the non-equilibrium Green’s function (NEGF) method, with the results depicted in Figure 7. In these results, the transmission spectra are shown to be completely compatible with the calculated band structures. By changing the number of subbands in an energy range, the transmission coefficient also changes. Both the energy bandgap and the transport bandgap (TBG) are completely consistent with each other. For both tensile and compressive applied strains, the bandgap size shrinks, when compared to the pristine, non-strained structure.

By applying compressive strain (−6%) to the structure, the valence and conduction subbands move toward the Fermi energy and separate from each other, which causes the reduction of the energy bandgap, and consequently, the TBG reduces to the value of 0.45 eV. Separating the energy bands increases the length of the steps in the transmission spectrum for the studied energy range. The tensile strain (8%) causes the subbands at the Γ point to move toward the Fermi energy, meaning that the energy bandgap and consequently the TBG are reduced to 1.04 eV. Also, the behavior of the ASbNR in the presence of tensile strain is changed to a direct bandgap semiconductor.

**Figure 8:**
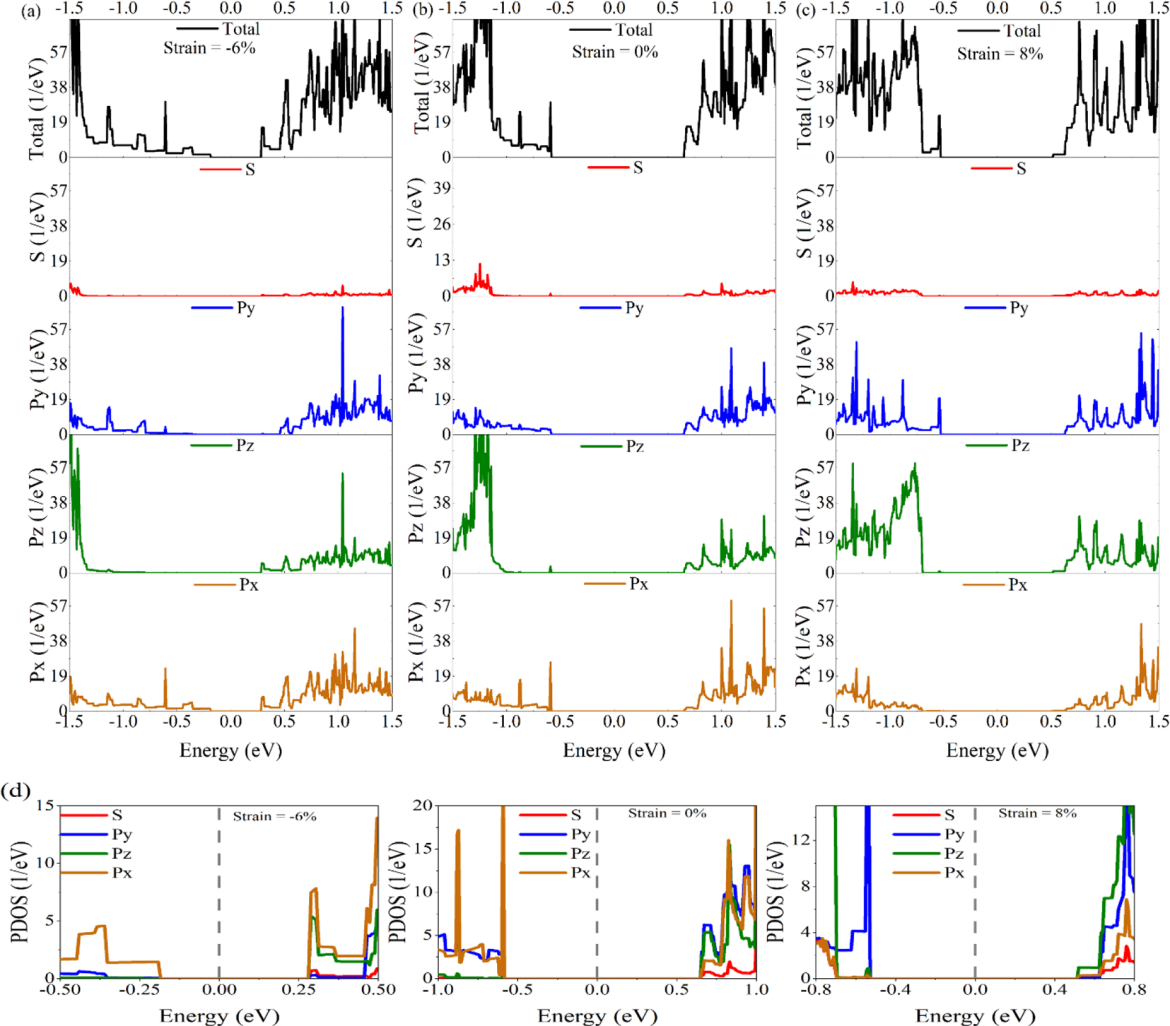
Density of state (DOS) for (a) 6% compressively strained, (b) unstrained, and (c) 8% tensile-strained 5-ASbNR. (d) Projected density of states (PDOS) of pristine and strained (−6%, 0%, and 8%) 5-ASbNR. The dashed-dotted line in panel (d) indicates the Fermi level (gray line).

For a deeper insight into the electronic and transport properties of nanoribbons, the calculated DOS and projected density of states (PDOS) of the pristine and strained structures of 5-ASbNR are compared in Figure 8. The energy gap of DOS is completely in agreement with the energy gap of the calculated bandstructure and transmission, which have been investigated previously. The PDOS of the pristine 5-ASbNR reveals that the VBM is formed by the hybridization of P_*x*_ and P_*y*_ orbitals, while the role of the P_*x*_ orbital is more dominant than the P_*y*_ orbital. The CBM is formed by the P_*y*_ and P_*z*_ orbitals, while the role of the P_*y*_ orbital is more dominant than the P_*z*_ orbital. In the presence of compressive strain (−6%), the VBM is formed by the P_*x*_ orbital, while the CBM is formed by the hybridization of the P_*x*_ and P_*z*_ orbitals. In the case of tensile strain (8%), the contributions of the P_*y*_ and P_*z*_ orbitals are the most dominant for the formation of the VBM and CMB, respectively. The results of the calculation in determining the primary orbitals, which participate in the formation of the valence and conduction band edges, are compiled in Table 3.

**Table 3:** Contribution of Orbitals to Form the CBM and VBM

band edge	strain = −6%	strain = 0%	strain = 8%
CBM	P_*x*_ & P_*z*_ (P_*x*_ > P_*z*_)	P_*y*_ & P_*z*_ (P_*y*_ > P_*z*_)	P_*z*_
VBM	P_*x*_	P_*x*_ & P_*y*_ (P_*x*_ > P_*y*_)	P_*y*_

## Conclusions

In this manuscript, we present a theoretical investigation of the effect of uniaxial strain on the electronic and transport properties of armchair antimonene nanoribbons (ASbNRs). The results show that wider nanoribbons are more energetically stable than narrower ones. Moreover, all modeled ribbons are semiconductors with an indirect bandgap. The energy bandgap of ASbNRs is inversely proportional to the width of the ribbons. By decreasing the width from 5 nm to 1 nm, the bandgap (indirect) increases from 1.2 eV to 2.4 eV. The electronic properties of ASbNR with a width of 5 nm (5-ASbNR) have been tuned by applying uniaxial compressive and tensile strain (−9% to 11%). The bandgap of 5-ASbNR decreases, when compressive or tensile strain is applied. A sufficiently high compressive strain (−9%) can convert the semiconducting behavior to a semi-metallic one, while the transition from indirect to direct bandgap can be achieved by applying a tensile strain above 4%. By stretching the structure, the electron and hole effective masses were decreased and increased, respectively. Compressing the structure had the opposite effect on the effective masses of holes and electrons. The mobility of carriers is inversely proportional to the effective mass; therefore, by applying compressive and tensile strain, the hole and electron mobility can be increased, when compared to the pristine, non-strained structure.
